# Supplementary material for: Selection of Reference Genes in Siraitia siamensis and Expression Patterns of Genes Involved in Mogrosides Biosynthesis
Source: Plants (Basel). 2024 Sep 2;13(17):2449. doi: 10.3390/plants13172449 (PMC11396801; doi:10.3390/plants13172449)
Supplement: Supplementary file 1 [file plants-13-02449-s001.zip › plants-3114652-supplementary.pdf]

Supplement dataset

Selection of reference genes in *Siraitia siamensis* and expression patterns of genes involved in mogrosides biosynthesis

Additional files

Supplementary Table S1. Effect of different treatments of phytohormones of *S. siamensis*.

Treatment	NAA (mg/L)	IBA (mg/L)	activated carbon(mg/L)	Growth Status
C1	0.1	0.3	100	Seedlings showed no increase, with some dead plants
C2	0.1	0.5	300	The seedlings showed no increase and no root growth
C3	0.1	0.7	200	The seedlings showed no increase, the bud tips yellow
C4	0.3	0.3	300	The seedlings grew relatively slowly, with yellow leaves
C5	0.3	0.5	200	The seedlings grew normally and propagated rapidly
C6	0.3	0.7	100	The seedlings grew normally, Some pollution
C7	0.5	0.3	200	The seedlings grew slowly, Leaves dark green
C8	0.5	0.5	100	The seedlings partially browned, the bud tips yellow
C9	0.5	0.7	300	The seedlings partially browned, the bud tips yellow

Supplementary Table S2. Primer information for twelve candidate reference genes.

Gene	Gene ID		Primer sequence (5′ - 3′)	Product size (bp)
SsRPL-13	*Ribosomal protein L-13*	F	TGACGCAAGGCACCACAT	110
		R	ATACGCTTCCTCAAGAACCTCAT	
SsCDC6	*Cell division control protein 6*	F	CCGAGGCAATCACTGTAAGA	128
		R	GCGAGGCGATTTCCAATTC	
SsTIP41	*TIP41-like family protein*	F	CGGACGACAAGGAATTGAA	109
		R	TTGGAGGAGGTAAGAATGGA	
SstubB	*β-tubulin2*	F	GGCGACTCCGAACTTCAG	98
		R	TCCAGGCTCCAGATCCATAA	
SsGAPDH	*glyceraldehyde-3-phosphate dehydrogenase*	F	ACCACTGTCCATTCCATTACTG	110
		R	CCATTCCCGTCAACTTTCCA	
SstubA	*α-tubulin2*	F	ACTGGTGTCCTACTGGCTTCA	120
		R	CGGCTGTGCTGTTGCTTATCATA	
SsEF1α	*Elongation factor 1α*	F	ACTTCACATCGCAGGTTATCATC	132
		R	CCAGCATCACCGTTCT TCAAG	
SsNCBP2	*Nuclear cap-binding protein subunit 2*	F	GCTTGTGGATTATGGTACTGGTTC	96
		R	GGCGGTCATCATCTCGGTAG	
SsUBQC	*ubiquitin C*	F	ACCTGGTGCTCCGATTGC	115
		R	AGTTGCTTGCCTGCGAAGA	
SstubB3	*β-tubulin3*	F	GGAGGCGGAGAACTGTGAT	114
		R	GGTTGCGTTGTAAGGCTCAA	
SsPP2A	*Protein phosphatase 2A*	F	TCGCCAGATTGCTCGGTGTCTA	123
		R	ACAGCCTGATTCCAGTGACTCCT	
SsPcACO	*1-aminocyclopropane-1-carboxylate oxidase A*	F	CGGCATCATCCTTCTCTTC	127
		R	CCATTCGTTATCACCTCAAGT	

Supplementary Table S3. Stability analysis of five treatments by NormFinder.

Rank	Low-temperature treatment		High-temperature treatment		Ethylene treatment		Salicylic acid treatment		Methyl jasmonate treatment	
1	0.229	*UBQC*	0.181	*NCBP2*	0.244	*CDC6*	0.256	*CDC6*	0.276	*NCBP2*
2	0.264	*NCBP2*	0.306	*tubB3*	0.385	*NCBP2*	0.283	*NCBP2*	0.285	*CDC6*
3	0.298	*CDC6*	0.484	*EF1α*	0.434	*tubB3*	0.416	*tubB3*	0.409	*TIP41*
4	0.325	*tubB2*	0.508	*TIP41*	0.547	*UBQC*	0.442	*TIP41*	0.458	*GAPDH*
5	0.332	*tubA*	0.519	*PP2A*	0.551	*tubA*	0.481	*GAPDH*	0.472	*UBQC*
6	0.354	*TIP41*	0.522	*tubB2*	0.562	*PCACO*	0.49	*tubA*	0.579	*tubB3*
7	0.44	*PCACO*	0.543	*CDC6*	0.586	*TIP41*	0.549	*UBQC*	0.617	*EF1α*
8	0.509	*EF1α*	0.617	*UBQC*	0.604	*RPL-13*	0.561	*RPL-13*	0.633	*PCACO*
9	0.528	*PP2A*	0.663	*tubA*	0.616	*EF1α*	0.686	*PCACO*	0.705	*RPL-13*
10	0.53	*RPL-13*	0.696	*RPL-13*	0.694	*GAPDH*	0.726	*tubB2*	0.744	*tubA*
11	0.707	*GAPDH*	0.951	*PCACO*	0.98	*tubB2*	0.934	*EF1α*	0.876	*tubB2*
12	0.753	*tubB3*	0.97	*GAPDH*	1.005	*PP2A*	1.051	*PP2A*	1.259	*PP2A*

Supplementary Table S4. Stability analysis of five treatments by Delta Ct.

Rank	Low-temperature treatment		High-temperature treatment		Ethylene treatment		Salicylic acid treatment		Methyl jasmonate treatment	
1	0.51	*NCBP2*	0.65	*NCBP2*	0.67	*CDC6*	0.65	*CDC6*	0.71	*CDC6*
2	0.51	*UBQC*	0.68	*tubB3*	0.73	*NCBP2*	0.67	*NCBP2*	0.71	*NCBP2*
3	0.54	*CDC6*	0.73	*EF1α*	0.73	*tubB3*	0.71	*tubB3*	0.77	*TIP41*
4	0.55	*TIP41*	0.77	*CDC6*	0.78	*tubA*	0.72	*TIP41*	0.78	*GAPDH*
5	0.56	*tubB2*	0.78	*TIP41*	0.82	*RPL-13*	0.74	*tubA*	0.78	*UBQC*
6	0.56	*tubA*	0.79	*tubB2*	0.82	*EF1α*	0.76	*RPL-13*	0.85	*EF1α*
7	0.6	*PCACO*	0.79	*PP2A*	0.82	*UBQC*	0.77	*GAPDH*	0.85	*tubB3*
8	0.66	*EF1α*	0.82	*UBQC*	0.83	*PCACO*	0.81	*UBQC*	0.89	*PCACO*
9	0.67	*RPL-13*	0.85	*RPL-13*	0.85	*TIP41*	0.89	*PCACO*	0.9	*RPL-13*
10	0.68	*PP2A*	0.87	*tubA*	0.93	*GAPDH*	0.91	*tubB2*	0.93	*tubA*
11	0.81	*GAPDH*	1.07	*PCACO*	1.12	*tubB2*	1.03	*EF1α*	1.04	*tubB2*
12	0.86	*tubB3*	1.09	*GAPDH*	1.16	*PP2A*	1.15	*PP2A*	1.37	*PP2A*

Supplementary Table S5. The results of all comprehensive analysis using RefFinder.

Gene name	Low-temperature treatment			High-temperature treatment			Ethylene treatment			Salicylic acid treatment			Methyl jasmonate treatment		
Method	1	2	3	1	2	3	1	2	3	1	2	3	1	2	3
Delta CT	*UBQC*	*NCBP2*	*CDC6*	*NCBP2*	*tubB3*	*EF1α*	*CDC6*	*NCBP*	*tubB3*	*CDC6*	*NCBP*	*tubB3*	*NCBP*	*CDC6*	*TIP41*
bestkeeper	*EF1α*	*CDC6*	*tubB2*	*EF1α*	*NCBP2*	*CDC6*	*PP2A*	*CDC6*	*TIP41*	*CDC6*	*TIP41*	*PCACO*	*UBQC*	*tubB2*	*NCBP*
Normfinder	*UBQC*	*NCBP2*	*CDC6*	*NCBP2*	*tubB3*	*EF1α*	*CDC6*	*NCBP*	*tubB3*	*CDC6*	*NCBP*	*tubB3*	*NCBP*	*CDC6*	*TIP41*
Genorm	*TIP41*	*UBQC*	*NCBP2*	*EF1α*	*CDC6*	*UBQC*	*EF1α*	*RPL13*	*tubA*	*CDC6*	*tubA*	*RPL13*	*UBQC*	*RPL13*	*tubA*
Comprehensive	*UBQC*	*NCBP2*	*CDC6*	*EF1α*	*NCBP2*	*tubB3*	*CDC6*	*NCBP*	*PP2A*	*CDC6*	*NCBP*	*tubA*	*CDC6*	*NCBP2*	*TIP41*

Supplementary Table S6. Primer information for 14 target genes

Gene	E.C	Gene ID		Primer sequence (5′-3′)	Product size (bp)
SsAACT	2.3.1.9	*acetyl-CoA C-acetyltransferase*	F	ATCCTTCAGCATTCCATCA	146
			R	CAGCACAGAGTATCCAGTT	
SsHMGS	2.3.3.10	*hydroxymethylglutaryl-CoA synthase*	F	CCATAGCGAAGTCATTGTAA	147
			R	TTGTTATTGCTGGTGGTATG	
SsHMGR	1.1.1.34	*hydroxymethylglutaryl-CoA reductase*	F	CATTAGCAACCACACCATT	99
			R	GCACCTACTATCTCACTGAA	
SsMK	2.7.1.36	*mevalonate kinase*	F	AGAAGAAGAGCGTGAAGA	98
			R	GAAGGTGAAGACTGTTGAC	
SsPMK	2.7.4.2	*phosphomevalonate kinase*	F	ACCATCCACAACCATCTTA	110
			R	ATCGTTATGCTGCTCCAT	
SsMVD	4.1.1.33	*diphosphomevalonate decarboxylase*	F	CTTCAACACCACCTTCAC	109
			R	TTACTGCTCCGACAAGAA	
SsIPI	5.3.3.2	*Isopentenyl-diphosphate Delta-isomerase*	F	ACTTCATCGTCGTCATCA	89
			R	TACCTTCTCGGCTTCTTC	
SsGPS	2.5.1.10	*geranylpyrophosphate synthetase*	F	GGCAAGATGTTGGTGATG	133
			R	GTTCCGAGCAATGATGATAC	
SsFPS	2.5.1.21	*farnesyl pyrophosphate synthetase*	F	CAACTCTGCTAACTTCTCTTC	140
			R	GGACCATTAGACATCCTGAT	
SsSQS	1.14.14.17	*squalene synthase*	F	TGCTTCCGACAACTTATTC	82
			R	ATCTATGGTAGGTGCTGTT	
SsSQE	1.14.19.-	*squalene epoxidase*	F	CTGACAACTTCCTCCACAT	89
			R	GTCGTCCACACCTTCAAT	
SsCS	5.4.99.33	*Cucurbitadienol synthase*	F	TGAACTTACCACAGCAATG	87
			R	AAGGATGGAAGTGATAGTCT	
SsEPH		*Epoxide hydrolase*	F	CTTCACATCTGCCACTTC	133
			R	CATTCCTCTCGGTCGTAT	
SsCYP		*Cytochrome P450*	F	CATTGACATTCCGAACTACA	134
			R	TGGTATTGCTGCTGAAGA	

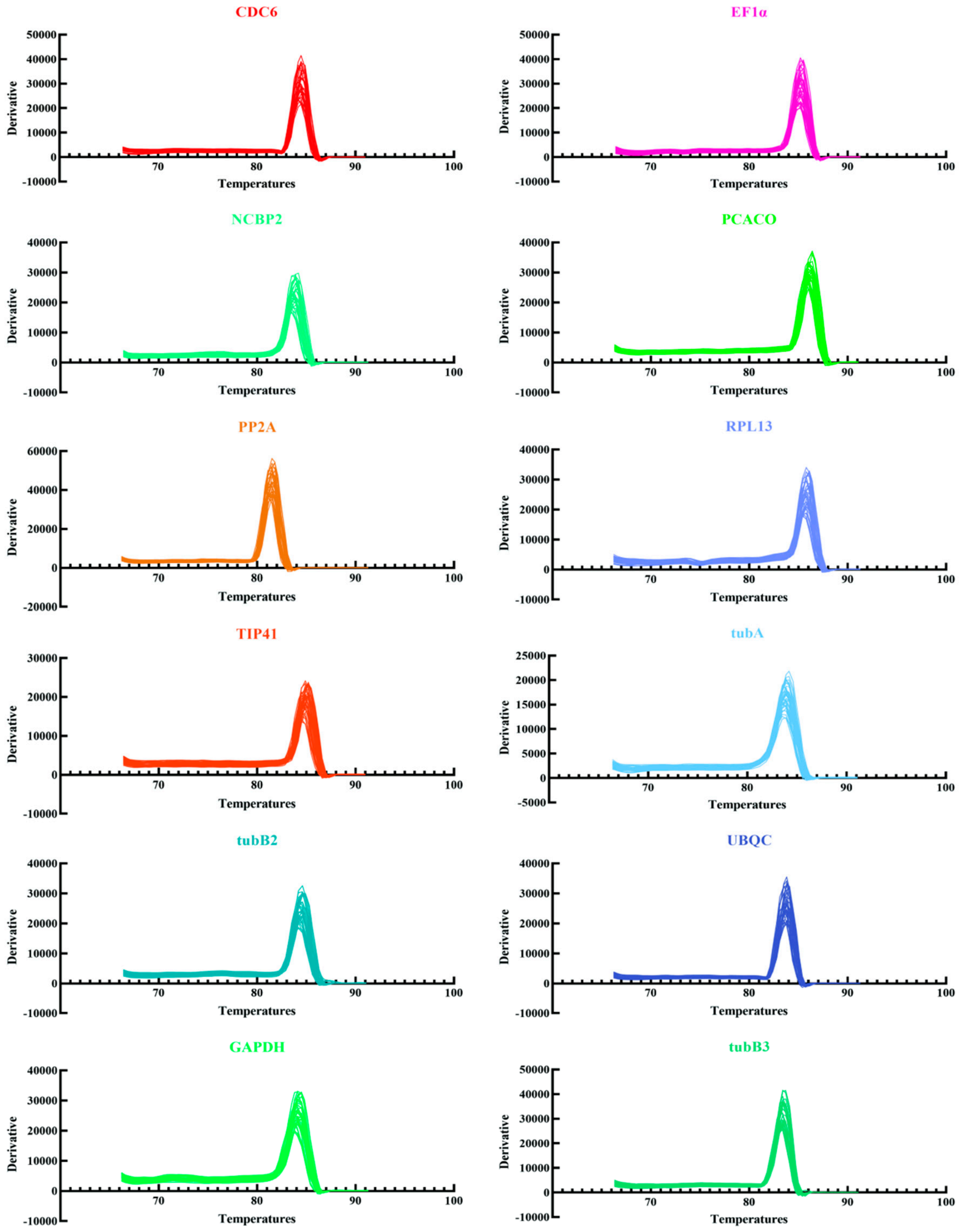

Supplementary Figure S1. RT- qPCR melting curves of twelve reference genes.

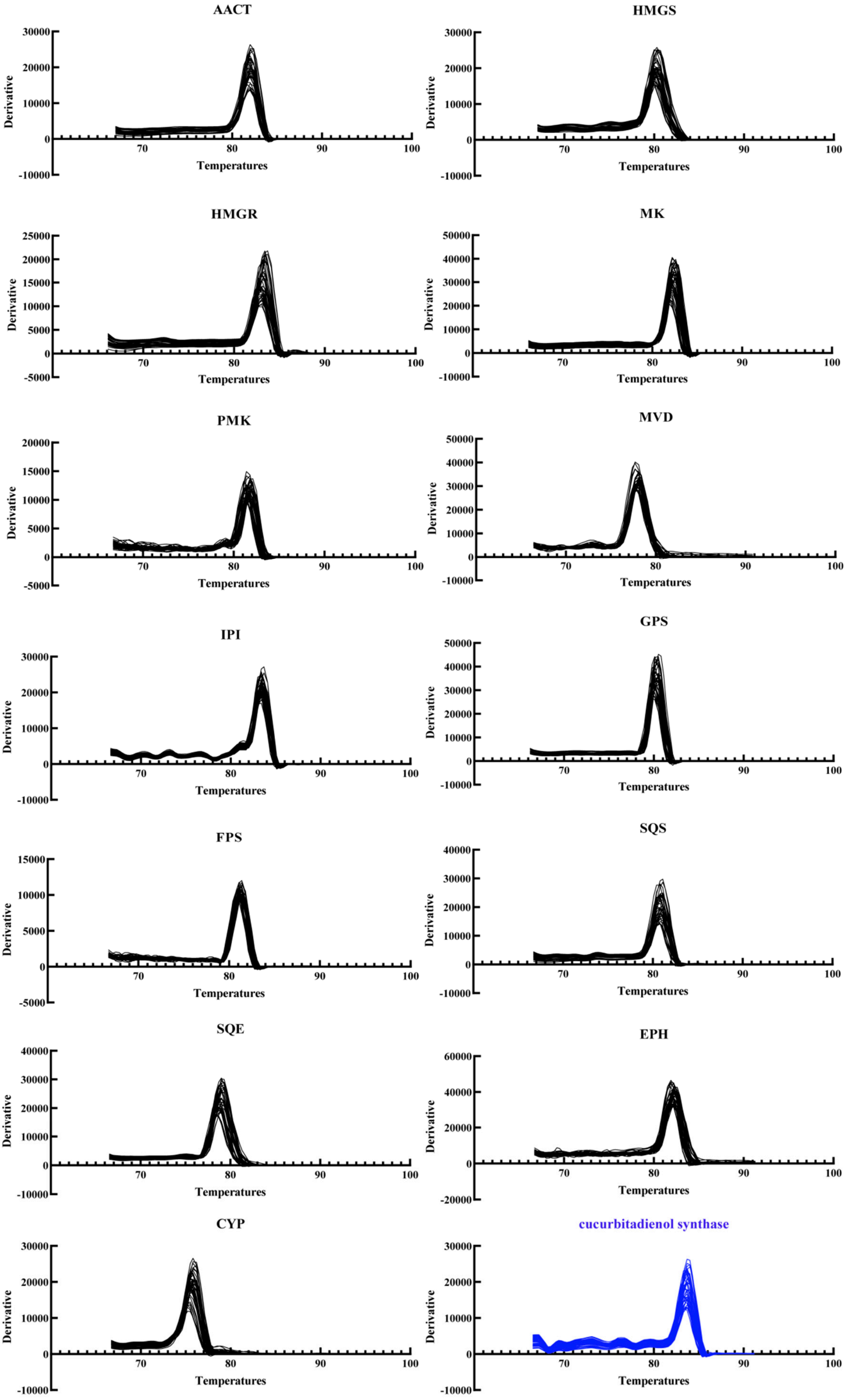

Supplementary Figure S2. RT- qPCR melting curves of fourteen mogrosides synthesis pathway genes.
